# Se-enriched yeast improves meat quality through glycerophospholipid metabolism in finishing pigs: insights from a multi-omics analysis

**DOI:** 10.1186/s40104-026-01446-3

**Published:** 2026-07-05

**Authors:** Xiangqi Qiu, Panpan Lu, Mingfei Xiao, Sumei Zeng, Li Li, Haitao Yu, Aihua Deng, Min Zhu, E Xu, Xiangfang Zeng

**Affiliations:** 1 Key Laboratory of Animal Genetics, Breeding and Reproduction in the Plateau Mountainous Region, Ministry of Education/Institute of Animal Nutrition and Feed Science, College of Animal Science, Guizhou University, Guiyang, 550025 China; 2 Guangdong Provincial Key Laboratory of Animal Nutrition Control/Guangdong Laboratory for Lingnan Modern Agriculture, State Key Laboratory of Swine and Poultry Breeding Industry, College of Animal Science, South China Agricultural University, Guangzhou, 510642 China; 3 State Key Laboratory of Animal Nutrition and Feeding, College of Animal Science and Technology, China Agricultural University, Beijing, 100193 China

**Keywords:** Lipidomic, Meat quality, Pigs, Se-enriched yeast, Transcriptomic

## Abstract

**Background:**

The improvement of meat quality by selenium (Se) supplementation has been well documented; however, the effects and underlying mechanisms by which Se-enriched yeast modulates meat quality remain unclear. This study aimed to investigate the effects of Se-enriched yeast on meat quality and its molecular characteristics in finishing pigs. All pigs were fed a Se-deficient diet (SeD) or the same diet supplemented with 0.3, 1, 3, or 5 mg Se/kg (CT, SY1, SY3, and SY5).

**Results:**

Compared with SeD, Se-enriched yeast supplementation, especially SY3, improved marbling score, intramuscular fat content, antioxidant capacity, and fatty acid composition in the LD muscle (*P* < 0.05). Lipidomic analysis revealed that Se-enriched yeast supplementation altered the abundances of multiple lipid species, while transcriptomic analysis showed significant enrichment of pathways related to unsaturated fatty acid and glycerophospholipid metabolism (*P* < 0.05). Integrated analyses further identified glycerophospholipid metabolism as a key pathway associated with major lipids, including phosphatidylcholine (PC) and phosphatidylethanolamine (PE), along with regulatory genes glycerol-3-phosphate acyltransferase 3 (*GPAT3*) and phosphatidylserine decarboxylase (*PISD*). Correlation analysis indicated that stearoyl-CoA desaturase (*SCD*), elongation of very long chain fatty acids protein 4 (*ELOVL4*), and *GPAT3* play crucial roles in lipid synthesis.

**Conclusion:**

Se-enriched yeast supplementation at 3 mg/kg improved meat quality, primarily through the regulation of glycerophospholipid metabolism, enhancement of membrane stability, and lipid homeostasis. These findings offer mechanistic insights into the role of Se-enriched yeast and highlight its potential as an effective feed additive for improving meat quality in finishing pigs.

**Supplementary Information:**

The online version contains supplementary material available at 10.1186/s40104-026-01446-3.

## Introduction

Pork is one of the most widely consumed meats worldwide. However, pigs are highly susceptible to oxidative stress under intensive production systems, which can lead to protein and lipid oxidation, ultimately compromising meat quality. Notably, lipids are particularly prone to oxidative degradation [1], making them a critical target in the deterioration of meat quality.

Meat quality is closely associated with lipid composition, especially phospholipids, which are major structural components of muscle cell membranes and serve as reservoirs for polyunsaturated fatty acids (PUFA). These lipids play essential roles in determining meat flavor, tenderness, and nutritional value [2]. Disruption of phospholipid homeostasis can impair membrane integrity, mitochondrial function, and redox balance, thereby accelerating lipid oxidation and deteriorating meat quality. Emerging evidence indicates that micronutrient status can profoundly influence lipid metabolism and fatty acid profiles [3, 4]. Accumulating studies indicate that dietary supplementation with feed additives can improve meat quality by reducing oxidative damage and alleviating physiological stress in pigs [5–7].

Selenium (Se) is a vital trace element and plays a critical role in maintaining redox homestasis by regulating antioxidant systems, particularly via selenoproteins such as glutathione peroxidase (GSH-Px), and its deficiency can lead to oxidative damage and related pathological alterations [8, 9]. Consequently, appropriate dietary Se supplementation has been widely recognized as an effective strategy to enhance antioxidant capacity and improve meat quality [10]. Importantly, the biological efficacy of Se is highly dependent on its chemical form. Compared with inorganic Se, organic Se exhibits higher bioavailability, greater tissue retention, and lower toxicity, making it more effective in promoting Se deposition in tissue and improving meat quality [11]. Accordingly, increasing attention has been paid to organic Se supplementation, previous studies have demonstrated that dietary supplementation with organic Se can improve animal health and meat quality [12, 13]. However, the molecular basis for the effects of Se-enriched yeast on meat quality in finishing pigs remains unclear, particularly regarding lipid metabolism and phospholipid remodeling.

With the rapid development of high-throughput omics technologies, lipidomics and transcriptomics have become powerful tools for elucidating the molecular basis of meat quality. Lipidomics provides a comprehensive view of lipid composition and its alterations, while transcriptomics facilitates the identification of key genes and signaling pathways involved in lipid metabolism and oxidative stress responses [14, 15]. Importantly, combining these two approaches offers a more integrated perspective, enabling a better understanding of lipid–gene interactions and the metabolic pathways underlying meat quality traits in pigs [16].

Therefore, this study aimed to investigate the effects of Se-enriched yeast on meat quality, antioxidant capacity, fatty acid profile, and lipid composition in finishing pigs. Furthermore, an integrated lipidomic and transcriptomic approach was applied to elucidate the molecular basis of Se-mediated regulation of lipid and phospholipid metabolism in finishing pigs. This study provides mechanistic insights and a theoretical basis for the application of Se-enriched yeast as a functional feed additive to improve pork quality.

## Materials and methods

### Animals and experimental design

A total of sixty healthy crossbred male pigs (Duroc × Landrace × Yorkshire; 100 d; 54 ± 2 kg) were randomly assigned into five treatment groups, each consisting six replicates with two pigs per replicate. The pigs were fed Se-deficient diet (SeD) or the same diet supplemented with 0.3 (control, CT), 1.0 (SY1), 3.0 (SY3), or 5.0 (SY5) mg Se/kg in the form of Se-enriched yeast (Angel Yeast Co., Ltd., Yichang, China). The analyzed Se concentrations in the SeD, CT, SY1, SY3, and SY5 diets were 0.04, 0.34, 1.04, 3.04, and 5.04 mg/kg, respectively. Feed and water were available ad libitum. This study lasted for 84 d at the experimental farm of the Institute of Animal Science, Guangdong Academy of Agricultural Science. The basal diet was formulated according to the nutrient requirements of the NRC [17], and its composition is shown in Table S1.

### Sample collection

At the end of the feeding experiment, one pig with a body weight close to the average body weight of the corresponding group was selected from each replicate. Blood samples were collected from the external jugular vein. After a 12-h fast, pigs were slaughtered by electrical stunning followed by exsanguination. Samples of the longissimus dorsi (LD) muscle, liver, and kidney were collected post-mortem, immediately frozen in liquid nitrogen, and then stored at −80 °C for subsequent analysis.

### ICP-MS analysis

Se content was measured using an inductively coupled plasma mass spectrometer (ICP-MS; 7900, Agilent, Santa Clara, CA, USA). Before analysis, the instrument was preheated for 30 min and optimized with a tuning solution to improve sensitivity and reduce oxide formation and doubly charged ion interference. Serum, LD muscle, liver, and kidney samples were digested with nitric acid and hydrogen peroxide, and the digests were diluted with ultrapure water to obtain sample solutions. The standard working solution, blank and sample solutions were then analyzed together with an online germanium (Ge) internal standard. Se concentrations were calculated from the ratio of Se to the internal standard signal using a calibration curve.

ICP-MS parameters were as follows: helium flow rate, 4 mL/min; radio frequency power, 1,500 W; plasma gas flow rate, 15 L/min; carrier gas flow rate, 0.8 L/min; auxiliary gas flow rate, 0.4 L/min; atomization chamber temperature, 2 °C; sample uptake rate, 0.3 r/s; sampling depth, 10 mm; acquisition mode, peak jump (spectrum); integration time, 0.3 s; analysis mode, collision/reaction cell.

### Antioxidant enzyme and malondialdehyde (MDA) analysis

The activities of total superoxide dismutase (T-SOD), catalase (CAT), total antioxidant capacity (T-AOC), and GSH-Px, as well as MDA content, were measured in LD muscle using commercially available kits (Nanjing Jiancheng Bioengineering Institute, Nanjing, China) according to the manufacturers' instructions.

### Meat quality

The pH values and meat color of LD muscle were determined at 45 min and 24 h post-mortem. Muscle pH was detected at depth of 1 cm using an OPTO-STAR equipment (Matthäus, Nobitz-Klausa, Germany). Meat color parameters, including lightness (L*), redness (a*), and yellowness (b*) were measured at three locations on the LD muscle surface using a Chroma Meter (CR-300 Minolta, Osaka, Japan). Drip loss of LD muscle was measured as previously described [18]. Briefly, 50 g of LD muscle was taken and then suspended in a sealed plastic bag at 4 °C for 24 h, blotted dry with filter paper, and reweighed to calculate the drip loss percentage. For shear force determination, LD muscle samples were vacuum-packed and heated in a water bath at 80 °C until the internal temperature reached 70 °C, then cooled to 4 °C. Three cores were obtained parallel to the muscle fiber orientation and sheared using a texture analyzer (C-LM3B; Harbin, China). Shear force was expressed in Newtons (N). Marbling scores of LD muscle were evaluated according to the color scale ranging from 1 to 5. Intramuscular fat (IMF) content was determined from dried samples using a Soxhlet extraction system according to a previously described method [19].

### Determination of fatty acid composition

Fatty acid composition was determined as previously described [20]. Briefly, fatty acids were extracted and derivatized to fatty acid methyl esters (FAMEs) using acetyl chloride-methanol, with methyl undecanoate as the internal standard. FAMEs were analyzed using a 7890B gas chromatograph (Agilent Technologies, CA, USA), equipped with a HP-88 fused capillary column (100 m × 0.25 mm ×0.2 μm). Nitrogen gas was used as the carrier gas at a flow rate of 2 mL/min. The injector and detector temperature were set at 270 and 280 °C, respectively. The column temperature program was as follows: initial temperature at 100 °C for 13 min, increased to 180 °C at 10 °C/min and held for 10 min, then increased to 200 °C at 1 °C/min and held for 20 min, and finally raised by 30 °C/min to 230 °C for 5 min. Fatty acids were identified by comparing the retention times of FAME peaks with those of known standards.

### Lipidomic analysis

#### Lipid extraction

Lipids were extracted using a previously described method with minor modifications [21]. Briefly, 40 mg of LD muscle was mixed with 200 μL of ultrapure water and 280 μL of methanol, followed by the addition of 800 μL of methyl tert-butyl ether. The mixture was sonicated at 4 °C for 20 min and kept at room temperature for 30 min. After centrifugation at 10 °C and 14,000 × *g* for 15 min, the upper organic phase was collected and dried under a nitrogen stream. The dried extracts were reconstituted in 200 μL of isopropanol/acetonitrile (9:1, v/v), vortexed, and centrifuged for 15 min, the resulting supernatant was then subjected to LC–MS/MS analysis.

#### Lipidomic analysis

Lipidomic analysis was performed using ultra-high performance liquid chromatograph (UHPLC) system (Nexera LC-30A, Shimadzu, Kyoto, Japan) coupled with a Q Exactive mass spectrometer (Thermo Fisher Scientific, San Jose, CA, USA). Lipids were separated on a CSH C18 column maintained at 45 °C with a flow rate of 300 μL/min. The mobile phases consisted of (A) acetonitrile/water (6:4, v/v) containing 10 mmol/L ammonium formate and 0.1% formic acid, and (B) acetonitrile/isopropanol (1:9, v/v) with 10 mmol/L ammonium formate and 0.1% formic acid. The gradient elution program was as follows: 30% B for 2 min, increased linearly to 100% B over 23 min, returned to 30% B within 1 min, and equilibrated for 9 min. Mass spectrometry analysis was conducted in both positive and negative electrospray ionization (ESI) modes. The ESI conditions were set as follows: heater temperature, 300 °C; sheath gas flow rate, 45 arbitrary units; auxiliary gas flow rate, 15 arbitrary units; sweep gas flow rate, 1 arbitrary unit; spray voltage, 3.0 kV (positive) or −2.8 kV (negative); capillary temperature, 350 °C; S-Lens RF Level, 50%; and MS1 scan ranges, *m/z* 200–1,800. Data-dependent acquisition methods were used for MS/MS analyses.

#### Data processing and analysis

Lipidomics data were processed by LipidSearch software (version 4.0; Thermo Fisher Scientific, San Jose, CA, USA) for peak identification, extraction, alignment, and quantification.

Principal component analysis (PCA), orthogonal partial least squares discriminant analysis (OPLS-DA) and hierarchical clustering heatmap analysis were conducted to assess differences among groups. Lipids with a variable importance in projection (VIP) value > 1 and *P* < 0.05 were considered as differentially expressed lipids (DELs). These were visualized using volcano plots generated in R package (version 3.4.3).

### Transcriptomic analysis

#### RNA extraction and transcriptomic data analysis

Total RNA from LD muscle was extracted using RNA Isolater Total RNA Extraction Reagent (Vazyme, Nanjing, China). RNA quality was evaluated using an Agilent Bioanalyzer (Agilent Technologies, CA, USA). After cDNA library constructing, transcriptome sequencing was performed on an Illumina platform with a read length of 150 bp paired-end. Quality control was performed using FastQC (version 0.11.7). Gene expression level was normalized via analyzing fragments per kilobase of transcript per million mapped reads (FPKM). Differentially expressed genes (DEGs) were screened by false discovery rate (FDR) < 0.05 and |Log_2_ fold chang| > 1 using DESeq2 package (1.24.0), Functional enrichment analysis of DEGs was performed based on the Kyoto Encyclopedia of Genes and Genomes (KEGG) using a ClusterProfiler R package (3.18.1).

#### Quantitative real-time PCR

To validate the DEGs identified by transcriptome analysis, quantitative real-time PCR (qRT-PCR) was employed. Primer sequences are provided in Table S2. The housekeeping gene was glyceraldehyde-3-phosphate dehydrogenase (*GAPDH*), and the relative expression levels of the targeted genes were determined by the 2^−ΔΔCt^ method [22].

### Integrated analysis of lipidomic and transcriptomic

The DELs and DEGs were used to conduct a joint KEGG pathway analysis using MetaboAnalyst 5.0.

### Protein extraction and Western blot

Protein from LD muscle samples was extracted using RIPA lysis buffer (Solarbio, China). The total protein concentration was detected using a bicinchoninic acid (BCA) assay kit (Beyotime, China). Equal amounts of proteins were separated through sodium dodecyl sulfate–polyacrylamide gel electrophoresis (SDS-PAGE) and transferred onto polyvinylidene difluoride (PVDF) membranes, which were then blocked with 5% skimmed milk for 2 h. The membranes were incubated overnight at 4 °C with primary antibodies against GAPDH (60004-1-Ig), glycerol-3-phosphate acyltransferase 3 (GPAT3; 20603-1-AP), and phosphatidylserine decarboxylase (PISD; 16401-1-AP) (Proteintech, China). After washing with TBST, the membranes were incubated with secondary antibody for 2 h, and washed again. Proteins bands were visualized using enhanced chemiluminescence (ECL; Biosharp, China), and band intensities were quantified by Image J software.

### Statistical analysis

All data were analyzed using SPSS software (version 22.0; IBM, Armonk, NY, USA). Differences among groups were analyzed using one-way ANOVA followed by Tukey’s post hoc test. Normality and homogeneity of variance were checked using the Shapiro–Wilk test and Levene’s test, respectively. Statistical significance was defined as *P* < 0.05. Data are presented as mean ± standard error of the mean (SEM). Figures were prepared using GraphPad Prism software (version 8.0). Pearson’s correlation analysis was performed to evaluate the relationships between DELs and DEGs. A correlation network was constructed and visualized using Cytoscape software (version 3.4.0).

## Results

### Se-enriched yeast supplementation affects meat quality in finishing pigs

**Fig. 1:**
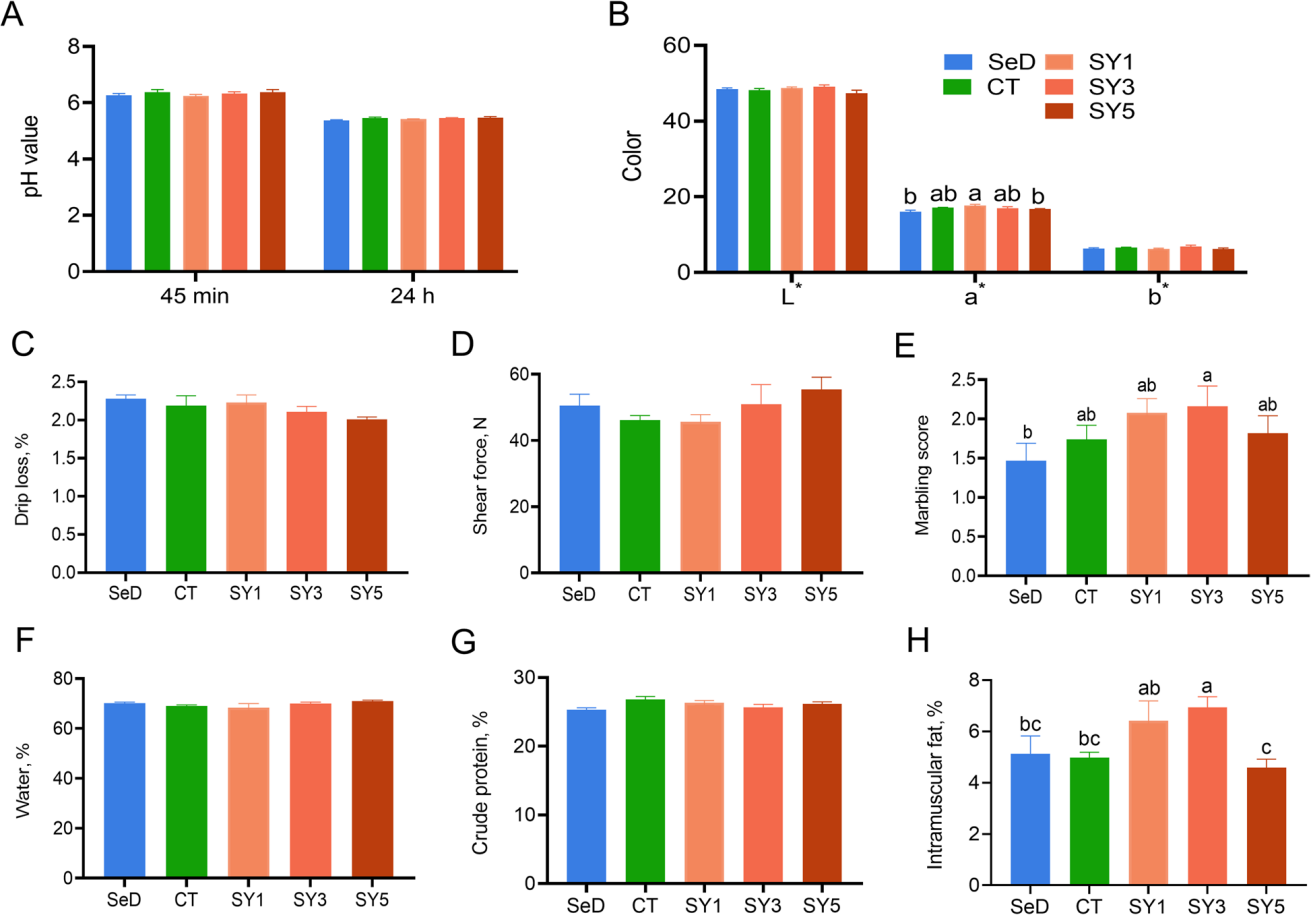
Effects of dietary Se-enriched yeast supplementation on meat quality in the longissimus dorsi (LD) muscle of finishing pigs. L*, lightness; a*, redness; b*, yellowness. SeD, Se-deficient group; CT, control group (basal diet); SY1, Se-enriched yeast group (basal diet +1 mg Se/kg); SY3, Se-enriched yeast group (basal diet + 3 mg Se/kg); SY5, Se-enriched yeast group (basal diet + 5 mg Se/kg). Data were presented as mean ± SEM, *n* = 6. Different letters indicate significant differences among groups (*P* < 0.05)

As shown in Fig. 1, the a* value of LD muscle was significantly higher (*P* < 0.05) in the SY1 group compared to the SeD and SY5 groups (Fig. 1B). The marbling score of LD muscle was significantly increased (*P* < 0.05) in the SY3 group than in the SeD group (Fig. 1E). The IMF content was significantly increased (*P* < 0.05) in the SY3 group compared with the SeD, CT, and SY5 groups (Fig. 1H). There was no significant difference in pH value, drip loss, shear force, water, and crude protein among groups (Fig. 1A, C, D, F, and G).

### Se-enriched yeast supplementation affects Se concentrations and redox status

**Fig. 2:**
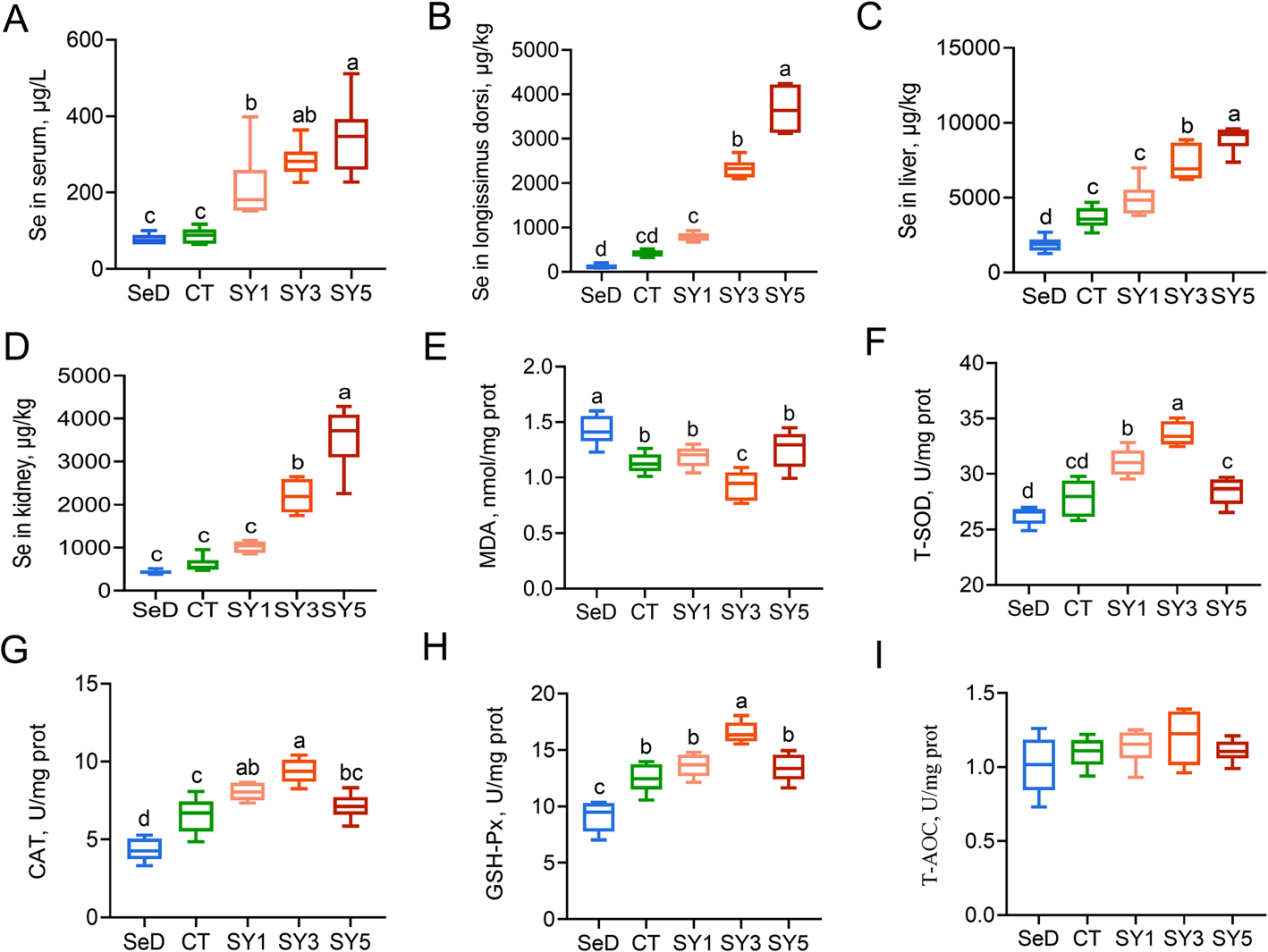
Effects of dietary Se-enriched yeast supplementation on Se contents in multiple tissues and antioxidant capacity in the longissimus dorsi (LD) muscle of finishing pigs. MDA, Malondialdehyde; T-SOD, Total superoxide dismutase activity; CAT, Catalase; GSH-Px, Glutathione peroxidase activity; T-AOC, Total antioxidant capacity. SeD, Se-deficient group; CT, Control group (basal diet); SY1, Se-enriched yeast group (basal diet +1 mg Se/kg); SY3, Se-enriched yeast group (basal diet + 3 mg Se/kg); SY5, Se-enriched yeast group (basal diet + 5 mg Se/kg). *n* = 6. Different letters indicate significant differences (*P* < 0.05)

Compared with the SeD and CT groups, Se concentrations in serum, LD muscle, liver, and kidney were significantly higher (*P* < 0.05) in both the SY3 and SY5 groups (Fig. 2A–D). In addition, Se concentrations in the serum, LD muscle, and liver were significantly higher (*P* < 0.05) in the SY1 group than in the SeD group, whereas no significant difference was observed in the kidney (*P* > 0.05) (Fig. 2A–D).

Compared with the SeD group, dietary supplementation with Se-enriched yeast (CT, SY1, SY3, SY5) significantly decreased (*P* < 0.05) MDA content and increased (*P* < 0.05) activities of T-SOD, CAT, and GSH-Px in LD muscle (Fig. 2E–H). Notably, T-SOD, CAT, and GSH-Px activities showed a positive dose–response relationship with increasing Se-enriched yeast supplementation from CT to SY3, but were decreased (*P* < 0.05) in the SY5 group compared with SY3. In contrast, MDA content was lowest in the SY3 group, with no difference among the CT, SY1 and SY5 groups. Moreover, T-AOC activity did not differ among groups (Fig. 2I).

### Se-enriched yeast supplementation affects fatty acid profiles of LD muscle

The fatty acid profiles of LD muscle in different groups are presented in Table 1. Compared with the SeD and CT groups, monounsaturated fatty acid (MUFA) content was significantly increased (*P* < 0.05) in the SY1 and SY3 groups. Specifically, palmitoleic acid (C16:1) and *trans*-9-octadecenoic acid (C18:1 n-9t) were higher (*P* < 0.05) in the SY1 group than in the CT, SY3, or SY5 groups. And, pentadecenoic acid (C15:1) was higher (*P* < 0.05) in the SY3 group than in the SeD and CT groups, while 10‑*cis*‑nonadecenoic acid (C19:1, *cis*-10) was increased (*P* < 0.05) in the SY3 and SY5 groups compared to the CT group.

**Table 1:** Effects of dietary Se-enriched yeast supplementation on fatty acid profiles in Longissimus dorsi (LD) muscle of finishing pigs

Fatty acids	Treatment					SEM	*P-*value
	SeD	CT	SY1	SY3	SY5		
C6:0	0.41	0.30	0.41	0.29	0.28	0.081	0.32
C8:0	1.22	0.81	1.07	0.99	0.82	0.235	0.42
C9:0	0.81^ab^	0.75^b^	1.19^a^	1.27^a^	0.88^bc^	0.148	0.00
C10:0	3.7	2.85	4.72	3.36	1.82	1.170	0.23
C11:0	0.05	0.08	0.07	0.07	0.07	0.020	0.71
C12:0	3.17	2.83	3.73	3.22	2.77	0.316	0.08
C14:0	28.27	26.79	33.66	28.56	25.65	2.808	0.12
C15:0	3.00	3.21	3.35	3.26	3.51	0.360	0.71
C16:0	716.67^b^	674.97^ab^	746.49^a^	751.75^a^	749.06^a^	23.80	0.04
C17:0	11.98	11.73	12.06	12.14	12.43	1.502	0.99
C18:0	433.57	429.50	473.68	466.84	479.46	24.620	0.21
C19:0	3.58	4.16	3.58	3.60	4.12	0.621	0.76
C20:0	4.60	4.68	5.01	4.89	4.95	0.179	0.17
C21:0	4.99	4.86	4.94	4.98	4.93	0.093	0.65
C22:0	5.00	4.95	5.03	4.97	4.97	0.097	0.92
C23:0	9.38	9.13	9.27	9.38	9.26	0.181	0.63
C24:0	6.25	6.20	6.25	6.28	6.23	0.129	0.98
C14:1	2.44	2.32	2.48	2.35	2.19	0.152	0.39
C15:1	20.82^b^	21.48^b^	22.10^ab^	24.25^a^	23.71^ab^	2.125	0.00
C16:1	59.73^ab^	53.46^b^	70.07^a^	65.35^ab^	57.03^b^	6.507	0.04
C16:1 T	5.62	4.25	4.52	5.02	4.37	0.614	0.22
C18:1 n-9c	381.30	379.36	414.58	413.43	398.35	27.550	0.41
C18:1 n-9t	66.93^ab^	61.49^ab^	74.55^a^	57.00^b^	53.48^b^	5.858	0.03
C19:1(*cis*-10)	8.36^ab^	8.68^b^	8.82^ab^	9.49^a^	9.45^a^	0.369	0.04
C20:1(*cis*-11)	11.04	10.58	12.07	11.81	12.41	0.839	0.24
C22:1 n-9	6.96	6.40	6.27	6.30	6.90	0.646	0.71
C24:1	18.13	17.55	17.81	18.13	17.84	0.404	0.59
C16:2	5.46	5.37	5.43	5.53	5.38	0.096	0.47
C18:2 n-6c	596.21	586.25	609.96	601.60	580.15	43.640	0.22
C20:2	15.87^ab^	13.15^b^	16.54^a^	16.76^a^	17.54^a^	1.066	0.01
C22:2	8.93	9.92	10.39	10.90	14.83	3.071	0.41
C18:3 n-3	10.12^b^	9.37^ab^	11.13^a^	11.93^a^	9.30^ab^	0.629	0.04
C18:3 n-6	9.76	10.50	10.92	9.48	9.85	1.055	0.65
C20:3 n-3	28.73	26.86	48	49.67	46.43	19.03	0.06
C20:3 n-6	30.57	28.32	29.81	27.67	28.49	3.125	0.43
C20:4 n-6	99.40	110.97	110.91	95.13	97.05	15.62	0.75
C20:5 n-3	27.81^ab^	26.57^b^	31.97^ab^	32.67^a^	27.20^ab^	1.574	0.01
C22:6 n-3	104.40	111.92	93.73	81.81	118.15	34.85	0.84
SFA	1236.65	1187.16	1314.51	1305.85	1311.21	35.75	0.06
MUFA	581.33^c^	565.57^c^	633.27^a^	613.13^ab^	585.73^bc^	51.20	0.048
PUFA	955.36^b^	954.84^b^	998.03^a^	984.38^ab^	959.45^b^	73.04	0.019

*SeD* Se-deficient group, *CT* control group (basal diet), *SY1* Se-enriched yeast group (basal diet +1 mg Se/kg), *SY3* Se-enriched yeast group (basal diet +3 mg Se/kg), *SY5* Se-enriched yeast group (basal diet +5 mg Se/kg), *SFA* Saturated fatty acid, *MUFA* Monounsaturated fatty acid, *PUFA* Polyunsaturated fatty acid. Data were presented as means, *n* = 6. Different letters indicate significant differences (*P* < 0.05)

Compare with the SeD, CT, and SY5 groups, PUFA content was higher (*P* < 0.05) in the SY1 group. Specifically, eicosadienoic acid (C20:2) was higher (*P* < 0.05) in all Se‑enriched yeast treatment groups compared with the CT group, whereas eicosapentaenoic acid (EPA, C20:5 n-3) was increased (*P* < 0.05) in the SY3 group. Similarly, α-linolenic acid (ALA, C18:3 n-3) was higher (*P* < 0.05) in the SY1 and SY3 groups than in the SeD group.

In addition, nonanoic acid (C9:0) in the SY1 and SY3 groups was significantly higher (*P* < 0.05) than in the CT and SY5 groups. Palmitic acid (C16:0) was increased (*P* < 0.05) in all Se‑enriched yeast treatment groups than in the SeD group.

### Optimal Se supplementation remodels the lipidomic profiles of LD muscle

**Fig. 3:**
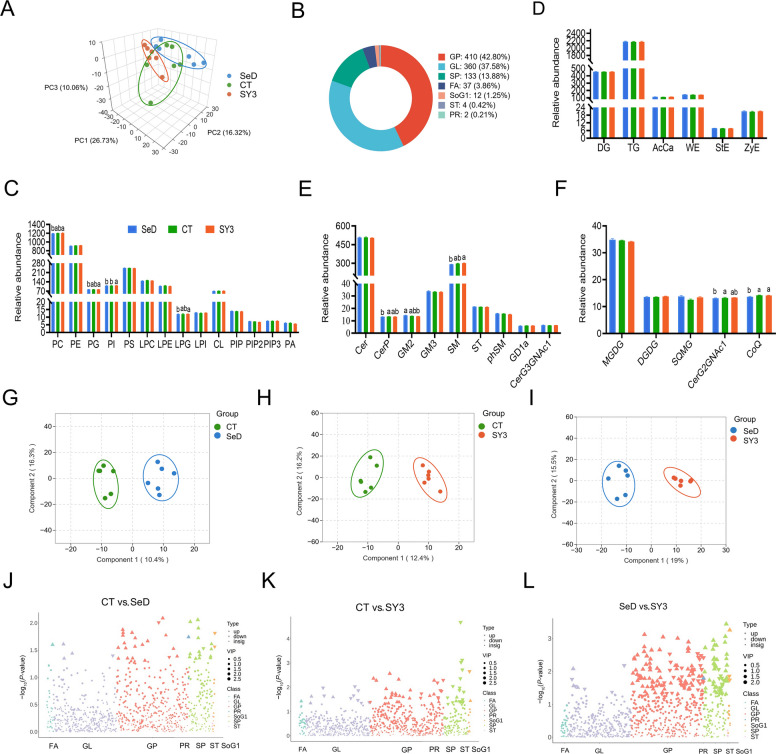
Lipidomic analysis of longissimus dorsi (LD) muscle in CT, SeD, and SY3 groups. **A** Principal component analysis (PCA) of lipidomic profiles. **B** Distribution of seven lipid classes based on the number of identified lipid species within each class. **C–F** Relative abundance of 34 lipid subclasses. Data were presented as mean ± SEM, *n* = 6. Different letters indicate significant differences (*P* < 0.05). **G–I** Orthogonal partial least squares discriminant analysis (OPLS-DA) plots of lipidomic profiles in three pairwise comparisons. **J–L** Volcano plots of differentially expressed lipids (DELs; VIP > 1, *P* < 0.05) in three pairwise comparisons. SeD, Se-deficient group; CT, control group (basal diet); SY3, Se-enriched yeast group (basal diet + 3 mg Se/kg)

Based on the above results, 3.0 mg/kg Se-enriched yeast was selected for lipidomic analysis due to its greater effects on meat quality, antioxidant capacity, and fatty acid composition. Lipidomic analysis of LD muscle was therefore performed in the SeD, CT, and SY3 groups. PCA showed a clear separation between the SeD and SY3 groups, while the CT group partially overlapped with both, indicating that Se status markedly influenced the overall lipid profile of LD muscle (Fig. 3A).

A total of 958 lipid species were identified and classified into seven classes, including 410 glycerophospholipids (GP), 360 glycerolipids (GL), 133 sphingolipids (SP), 37 fatty acyl class (FA), 12 glucosylsphingosine (GlcSph), 4 sterol lipids (ST), and 2 prenol lipids (PR) (Fig. 3B, Table S3). The relative abundance of GP, PR, and SP was significantly higher (*P* < 0.05) in CT and/or SY3 groups than in the SeD group (Fig. S1E–G), and no differences were found in other lipid species among groups (Fig. S1C, D, H, and I). Lipid species were further classified into 34 subclasses (Fig. S1B), among which major phospholipids, including phosphatidylcholine (PC), phosphatidylglycerol (PG) and phosphatidylinositol (PI), were significantly increased in the SY3 group compared with the SeD and/or CT groups (*P* < 0.05). In addition, coenzyme Q (CoQ) was also increased (*P* < 0.05) in the CT and SY3 groups. The abundances of lipid species are presented in Fig. 3C–F.

To investigate lipid alterations associated with Se status, differential lipid analysis was performed across the three groups. As shown in Fig. S1, OPLS-DA revealed clear group separation with robust model validation (R^2^Y and Q^2^) (Fig. 3G–I; Fig. S1J–L). Volcano plots identified numerous DELs (VIP > 1, *P* < 0.05) (Fig. 3J–L). Specifically, 44 DELs (6 upregulated and 38 downregulated) were identified in the CT vs. SeD comparison, 69 DELs (31 upregulated and 38 downregulated) were detected in the CT vs. SY3 comparison, and 176 DELs (123 upregulated and 53 downregulated) were observed in the SeD vs. SY3 comparison (Tables S4–S6). The top 10 representative DELs are presented in Fig. S1M–O. Notably, these DELs were mainly enriched in phospholipids, particularly PC, PE, indicating a marked remodeling of membrane lipid composition following Se-enriched yeast supplementation.

### Optimal Se supplementation affects the transcriptomic profiles of LD muscle

**Fig. 4:**
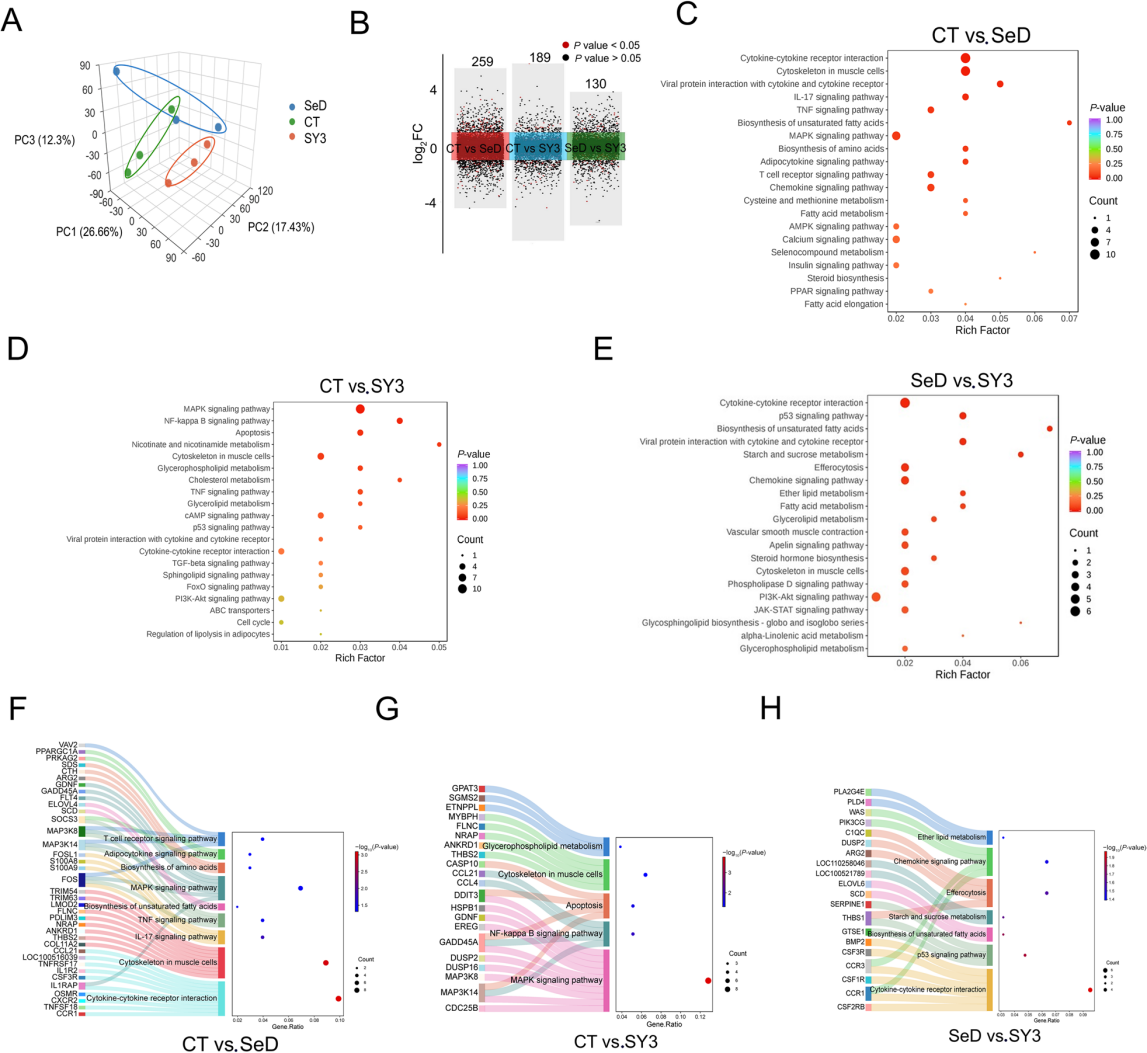
Transcriptomic analysis of longissimus dorsi (LD) muscle in SeD, CT, and SY3 groups. **A** Principal component analysis (PCA) analysis of gene profiles. **B** Volcano plots of differentially expressed genes (DEGs; Log_2_ |(Fold change)| > 1 and *P* < 0.05) in three pairwise comparisons. **C–E** Bubble plots of KEGG enrichment analysis in three pairwise comparisons. **F**–**H** Sankey plots of significantly enriched KEGG pathway with their corresponding DEGs in three pairwise comparisons. SeD, Se-deficient group; CT, control group (basal diet); SY3, Se-enriched yeast group (basal diet + 3 mg Se/kg)

To further elucidate the molecular mechanisms underlying the effects of Se status on meat quality, transcriptomic analysis was performed in the SeD, CT, and SY3 groups (Fig. 4). PCA showed a clear separation of the SY3 group from the SeD and CT groups, while partial overlap was observed between SeD and CT groups (Fig. 4A). Consistently, the heatmap revealed distinct gene expression patterns across the three groups (Fig. S2A). A total of 259 DEGs (121 upregulated and 138 downregulated), 189 DEGs (93 upregulated and 96 downregulated), and 130 DEGs (49 upregulated and 81 downregulated) were identified in CT vs. SeD, CT vs. SY3, and SeD vs. SY3, respectively (Fig. 4B, Fig. S2B; Tables S7–S9).

Functional enrichment analysis of the DEGs identified in the three pairwise comparisons was performed using the KEGG database. The top 20 enriched pathways for three pairwise comparisons are shown in Fig. 4C–E. In the CT vs. SeD comparison, DEGs were mainly enriched in pathways associated with inflammation and immune responses, such as cytokine-cytokine receptor interaction, IL-17 signaling pathway, and TNF signaling pathway, as well as lipid metabolism-related pathways including biosynthesis of unsaturated fatty acids and adipocytokine signaling pathway (Fig. 4F and Table S10). In the CT vs. SY3 comparison, DEGs were primarily enriched in the mitogen-activated protein kinase (MAPK) signaling pathway, NF-κB signaling pathway, apoptosis, cytoskeleton in muscle cells, and glycerophospholipid metabolism (Fig. 4G and Table S11). In addition, in the SeD vs. SY3 comparison, DEGs were mainly enriched in pathways associated with immune and inflammatory response and lipid metabolism, including cytokine signaling pathways, fatty acid biosynthesis, and ether lipid metabolism (Fig. 4H and Table S12). Collectively, these results suggest that Se status modulates immune and inflammatory responses, metabolic processes, and muscle-related pathways in pigs.

To validate the RNA-seq results, four DEGs were selected for qPCR analysis. Compared with the CT group, ankyrin repeat domain 1 (*ANKRD1*) was upregulated, whereas bone morphogenetic protein 2 (*BMP2*) was downregulated in the SY3 group. In addition, retinaldehyde binding protein 1 (*RLBP1*) and filamin C (*FLNC*) were significantly upregulated in the SeD group (Fig. S2C–F). These qPCR results were consistent with the RNA-seq data.

### Integrated analyses reveal a key role of glycerophospholipid metabolism in the improvement of meat quality by Se-enriched yeast

**Fig. 5:**
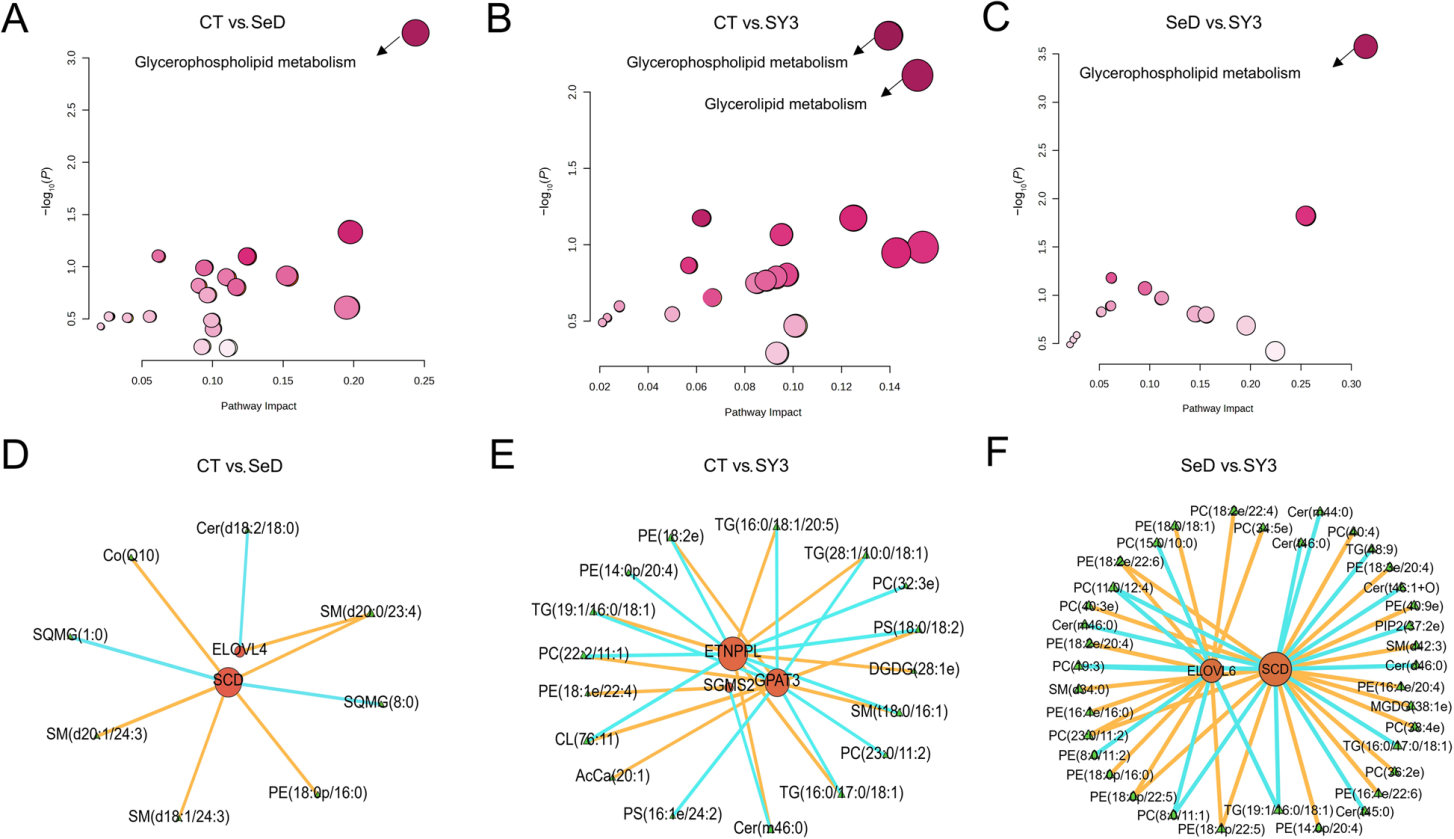
Integrated analysis of lipidomic and transcriptomic data in three pairwise comparisons. **A**–**C** Joint pathway analysis of DELs and DEGs in three pairwise comparisons. **D–F** Correlation network diagram of key DELs and DEGs in three pairwise comparisons. Blue lines represent negative correlations, and orange lines represent positive correlations (|*r*| > 0.9 and *P* < 0.05). Circles represent DEGs, and triangles represent DELs. SeD, Se-deficient group; CT, control group (basal diet); SY3, Se-enriched yeast group (basal diet + 3 mg Se/kg)

To further identify shared pathways between DELs and DEGs, a joint analysis of DELs and DEGs from three pairwise comparisons was performed using MetaboAnalyst. As shown in Fig. 5A, glycerophospholipid metabolism was significantly enriched pathway in the CT vs. SeD comparison, including two downregulated lipid species, PC and phosphatidylethanolamine (PE), and two downregulated genes, *GPAT3* and *PISD*. In the CT vs. SY3 comparison, glycerophospholipid metabolism and glycerolipid metabolism were identified as significantly enriched pathways. Specifically, glycerophospholipid metabolism was associated with increased levels of PC and the upregulation of sphingomyelin synthase 2 (*SGMS2*) and *GPAT3*, whereas glycerolipid metabolism was mainly associated with the upregulation of *GPAT3* and monoacylglycerol O-acyltransferase 2 (*MOGAT2*) (Fig. 5B). Moreover, in the SeD vs. SY3 comparison, three upregulated lipid species (PC, PE, and phosphatidyl-L-serine) and a downregulated gene (phospholipase D family member 4, *PLD4*) were enriched in glycerophospholipid metabolism (Fig. 5C).

To investigate the relationships between lipid metabolites and genes under Se deficiency or Se-enriched yeast supplementation, correlation analysis between DEGs and DELs was performed across the three pairwise comparisons (|*r*|> 0.9). In the CT vs. SeD comparison, stearoyl-CoA desaturase (*SCD*) was significantly positively correlated with several lipid species including PE, SM, and sulfoquinovosylmonoacylglycerol (SQMG), but negatively correlated with SQMG (1:0). Elongation of very long chain fatty acids protein 4 (*ELOVL4)* was significantly positively correlated with SM (d20:0/23:4) and negatively correlated with ceramide (Cer, d18:2/18:0) (Fig. 5D and Table S13). In the CT vs. SY3 comparison, lipid species including PC, PE, and SM were significantly positively correlated with *GPAT3*, whereas ethanolamine-phosphate phospho-lyase (*ETNPPL*) was positively correlated with triglyceride (TG) species (Fig. 5E and Table S14). In the SeD vs. SY3 comparison, most lipid species (e.g., PC, PE, SM, and PG) were significantly positively correlated with *SCD* and elongation of very long chain fatty acids protein 6 (*ELOVL6*), whereas some lipid species including diacylglycerol (DG), digalactosyldiacylglycerol (DGDG), TG, and Cer were negatively correlated with *SCD* and *ELOVL6* (Fig. 5F and Table S15).

**Fig. 6:**
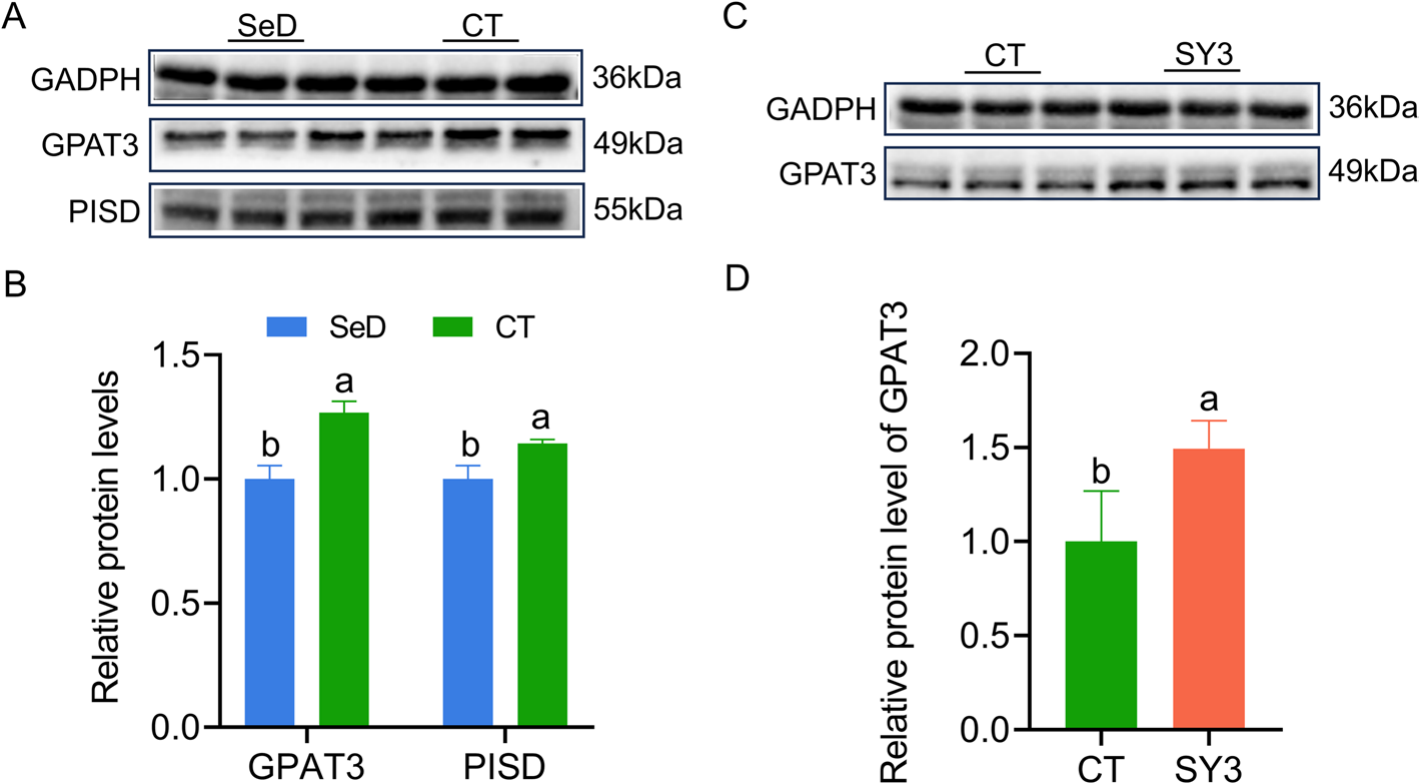
Protein expression validation of key lipid metabolism–related targets identified by integrated transcriptomic and lipidomic analyses in the longissimus dorsi (LD) muscle of finishing pigs. **A** Western blot analysis of glycerol-3-phosphate acyltransferase 3 (GPAT3) and phosphatidylserine decarboxylase (PISD) in SeD and CT groups. **B** Densitometric quantification of GPAT3 and PISD in SeD and CT groups. **C** Western blot analysis of GPAT3 in CT and SY3 groups. **D** Densitometric quantification of GPAT3 in CT and SY3 groups. SeD, Se-deficient group; CT, control group (basal diet); SY3, Se-enriched yeast group (basal diet + 3 mg Se/kg). Data were presented as mean ± SEM, *n* = 3. Different letters indicate significant differences (*P* < 0.05)

We selected representative candidate genes to further validate their protein expression levels by Western blot analysis in the LD muscle of finishing pigs. As shown in Fig. 6, the protein expression levels of GPAT3 and PISD were significantly higher (*P* < 0.05) in the CT group compared with the SeD group. In addition, GPAT3 protein abundance was increased (*P* < 0.05) in the SY3 group compared with the CT group. These results indicate that the expression patterns of the candidate genes are consistent at both the transcript and protein levels, thereby supporting the reliability of the integrated omics analysis.

## Discussion

Meat color is an important determinant of consumer preference. In this study, the a* value of LD muscle was increased in the SY1 group, consistent with a report showing that dietary Se supplementation can increase the a* value in pigs [12]. Another study reported no effect of dietary Se supplementation on meat color in pigs [23], which might be due to the differences in feeding regimes, breed, or Se sources. IMF content, reflected by marbling score, is a key indicator of pork quality and plays a critical role in sensory attributes such as flavor, juiciness, and tenderness [24]. In the current study, both marbling score and IMF content were higher in the SY3 group, consistent with previous findings that dietary organic Se supplementation increased IMF content in pigs [25]. These results indicate that Se-enriched yeast supplementation can effectively improve meat quality in finishing pigs.

Se-enriched yeast has higher bioavailability and lower toxicity compared to the inorganic Se [26]. In the present study, dietary supplementation with 3.0 and 5.0 mg/kg Se-enriched yeast increased the Se concentrations in serum, LD muscle, liver, and kidney. These data indicate that Se-enriched yeast improves systemic Se absorption, promotes Se deposition in LD muscle, and enhances Se utilization and excretion in kidney [27–29]. These findings were consistent with previous study showing that organic Se supplementation enhanced Se deposition [30].

Rapid growth of muscle tissues and meat storage processes are often accompanied by oxidative stress, which negatively affects meat quality, including both nutritional value and sensory attributes (color, taste, and odor). Adequate Se levels are essential for mitigating oxidative stress, whereas Se deficiency promotes oxidative stress and apoptosis [9, 31]. In this study, dietary supplementation with 3 mg/kg Se-enriched yeast significantly improved redox homeostasis in LD muscle compared with the SeD group, as evidenced by reduced lipid peroxidation (MDA) and increased activities of antioxidant enzymes (T-SOD, CAT, and GSH-Px). These results are consistent with previous studies, demonstrating that dietary Se supplementation enhances antioxidant defenses in pigs [9, 32]. No significant change in T-AOC was observed following Se-enriched yeast supplementation. This may be because T-AOC reflects the combined contribution of both enzymatic and non-enzymatic antioxidant systems, whereas Se primarily enhances enzymatic antioxidant defense, particularly Se-dependent GSH-Px. Notably, the antioxidant effect was most pronounced at 3.0 mg/kg, whereas supplementation at 5.0 mg/kg did not provide further improvement, although it remained within the safe range for dietary Se in pigs [33]. Taken together, these results suggest that supplementation with 3 mg/kg Se-enriched yeast may provide sufficient Se to support antioxidant enzyme synthesis and activity, thereby enhancing antioxidant capacity in the LD muscle and potentially contributing to improved meat quality.

Fatty acids are closely related to the nutritional value of meat. In this study, dietary supplementation with Se-enriched yeast altered fatty acid composition, including changes in some saturated fatty acid content in finishing pigs. Although its excessive intake has been associated with elevated cholesterol levels and increased cardiovascular risk, notably, Se-enriched yeast supplementation also significantly increased MUFA and PUFA contents, which are generally considered beneficial for human health. For example, omega-3 fatty acids have positive effects on growth, development, and overall health in both animals and humans [34]. Specifically, the increased levels of ALA and EPA observed in this study have been reported to reduce immune response, inflammation, and allergic reactions, and to lower the risk of cardiovascular diseases, diabetes, and cancer [35]. In addition to their nutritional benefits, MUFA and PUFA are susceptible to oxidation, contributing to the formation of desirable flavor compounds during cooking [36]. Consistently, a previous study reported that increased C18:1 content positively influenced pork flavor development [37], and another study demonstrated that Se supplementation altered fatty acid profile and enhanced meat aroma during cooking [38]. Overall, the improved fatty acid profile observed in this study may be attributed to the enhanced antioxidant capacity induced by Se-enriched yeast supplementation. These findings suggest that Se-enriched yeast may improve both the nutritional value and meat quality of finishing pigs.

Lipidomic profiling characterizes differences under varying Se statuses. A total of 1,179 lipid species were identified in LD muscle, comparable to the previous report in pork [39]. Suboptimal Se levels have been shown to disrupt lipid and one-carbon metabolism, thereby contributing to disease development [40]. In this study, Se deficiency markedly reduced multiple lipid classes, including GP, SP, and PR, as well as key lipid species such as PC, PG. Consistent with previous finding, Se insufficiency induced lipid metabolic reprogramming, particularly by suppressing phospholipid synthesis, including PE and PC [41]. Notably, adequate Se supplementation restored these lipid species in finishing pigs. GP is the major component of cellular membranes and is essential for cell growth, lipid metabolism, and signal transduction [42]. Among them, PC serves as a major reservoir of PUFAs and is closely associated with meat quality [43]. PG, a precursor of cardiolipin, is critical for mitochondrial membrane integrity and respiration function [44]. PI, although a minor membrane lipid, plays a key role in intracellular signaling [45]. SP participates in signal transduction and cell recognition [46]. Moreover, CoQ, a mitochondrial membrane component, functions as an antioxidant essential for mitochondrial function [47]. Notably, PG and CoQ have been identified as biomarkers associated with meat color and overall meat quality [4]. Overall, these lipidomic alterations suggest that Se plays a critical role in maintaining lipid metabolic homeostasis in LD muscle, thereby contributing to enhanced meat quality in finishing pigs. These lipidomic findings demonstrated that dietary optimal Se supplementation, especially Se-enriched yeast, improved lipid profile, including key membrane-stabilizing phospholipids and signaling lipids, which may underlie the observed improvements in oxidative stability and meat quality.

Transcriptomic analysis further revealed that gene profile was influenced by Se status in finishing pigs. Previous studies have consistently shown that dietary Se deficiency induces extensive transcriptomic reprogramming, particularly affecting pathways related to inflammation, immune dysregulation, and apoptosis, thereby impairing tissue homeostasis and organ function [41, 48]. Consistent with previous reports, Se deficiency in the present study activated multiple immune and inflammatory pathways in LD muscle, such as IL-17 signaling, TNF signaling, and T cell receptor signaling. In addition, pathways related to cytoskeletal organization, MAPK signaling, and amino acid biosynthesis were also enriched, which alters cell growth and morphogenesis. In contrast, Se-enriched yeast supplementation mainly regulated glycerophospholipid metabolism and starch and sucrose metabolism, along with pathways related to cytoskeletal organization, NF-κB signaling, p53 signaling, and MAPK signaling. Glycerophospholipids are essential components of muscle cell membranes and play critical roles in lipid deposition, membrane integrity, and lipid droplet formation [49]. Therefore, the regulation of glycerophospholipid metabolism by Se-enriched yeast may represent a key mechanism underlying Se-mediated lipid metabolic homeostasis in LD muscle.

Lipid metabolism is essential for cellular integrity and plays a pivotal role in determining meat quality. Integrated analyses of DELs and DEGs revealed that Se nutritional status regulates lipid metabolic pathways, particularly glycerophospholipid metabolism. Under Se-deficient conditions, glycerophospholipid metabolism was significantly disrupted, involving PC, PE, *GPAT3*, and *PISD*. Reduced *PISD* expression likely impaired PE synthesis, potentially compromising membrane integrity and subsequently suppressing *GPAT3* expression and TG synthesis, consistent with the role of *GPAT3* as a key regulator of lipid deposition [50, 51]. In contrast, Se-enriched yeast supplementation induced coordinated enrichment of glycerophospholipid and glycerolipid metabolic pathways, accompanied by upregulation of *GPAT3*, *MOGAT2*, *SGMS2*, and *ETNPPL*. MOGAT2 and GPAT3 are key enzymes promoting TG synthesis, lipid droplet formation, or phospholipid accumulation [52, 53]. SGMS2 mediates sphingomyelin synthesis and contributes to glycerophospholipid homeostasis [54]. Correlation analysis further revealed that *GPAT3* and *ETNPPL* were positively associated with TG and phospholipid species, supporting their roles in lipid anabolic processes. In addition, Se status also affected the biosynthesis of unsaturated fatty acids, such as *SCD* and *ELOVL4/6* genes known to positively correlate with lipid molecules. Notably, negative correlations between genes and lipid species may reflect compensatory regulatory mechanisms that limit excessive lipid deposition [55].

Collectively, these findings indicate that Se deficiency disrupts lipid metabolic homeostasis, whereas Se-enriched yeast supplementation promotes coordinated regulation of glycerophospholipid and fatty acid metabolism. This Se-dependent modulation of lipid pathways may contribute to IMF deposition and improved meat quality in finishing pigs.

## Conclusion

This study demonstrates that dietary supplementation with Se-enriched yeast improves meat quality including color, marbling score, IMF content, and fatty acid profile in finishing pigs through enhancing antioxidant capacity. More importantly, integrated lipidomic and transcriptomic analyses identified glycerophospholipid metabolism as a key regulatory underlying these improvements, where Se deficiency impaired this pathway by reducing key phospholipids (PC and PE) and downregulating associated genes (*GPAT3* and *PISD*), whereas Se-enriched yeast supplementation reversed these alterations by restoring phospholipid homeostasis and activating genes involved in lipid synthesis (e.g., *GPAT3* and *MOGAT2*). These findings extend the role of Se beyond its antioxidant function and suggest that Se may improve pork quality through the regulation of glycerophospholipid metabolism, providing a potential mechanistic basis for precision Se nutrition in pig production.

However, several limitations of this study should be acknowledged. First, this study focused only on LD muscle, and the effects of Se-enriched yeast on other tissues remain unclear. Second, although two selected genes were validated at the protein level by Western blot, the functional roles of the identified DEGs and DELs were not comprehensively verified in vitro or in vivo, and further studies will be conducted to elucidate their roles in regulating meat quality in pigs. From an economic perspective, the feasibility of large-scale application of high dose Se-enriched yeast supplementation requires further evaluation.

## Supplementary Information

Additional file 1: Table S1. Composition and nutrient contents of basal diet. Table S2. Primer used for quantitative real-time PCR. Table S3. Lipidomic profiles of LD muscle in SeD, CT, and SY3 groups. Table S4. DELs of LD muscle in CT vs. SeD. Table S5. DELs of LD muscle in CT vs. SY3. Table S6. DELs of LD muscle in SeD vs. SY3. Table S7. DEGs of LD muscle in CT vs. SeD. Table S8. DEGs of LD muscle in CT vs. SY3. Table S9. DEGs of LD muscle in SeD vs. SY3. Table S10. The top 20 KEGG pathways of LD muscle in CT vs SeD. Table S11. The top 20 KEGG pathways of LD muscle in CT vs. SY3. Table S12. The top 20 KEGG pathways of LD muscle in SeD vs. SY3. Table S13. Correlation analysis of LD muscle in CT vs. SeD. Table S14. Correlation analysis of LD muscle in CT vs. SY3. Table S15. Correlation analysis of LD muscle in SeD vs. SY3.

Additional file 2: Fig. S1. Lipidomic analysis of longissimus dorsi muscle in CT, SeD, and SY3 groups. Fig. S2. Transcriptomic analysis of longissimus dorsi in SeD, CT, and SY3 groups.

Additional file 3. The uncropped Western blot images.

## Data Availability

The data used and analyzed during the current study are available from the corresponding author on reasonable request.

## Abbreviations

a*: Redness value
ALA: α-linolenic acid
ANKRD1: Ankyrin repeat domain 1
b*: Yellowness value
BMP2: Bone morphogenetic protein 2
CAT: Catalase
DEGs: Differentially expressed genes
DELs: Differentially expressed lipids
DG: Diacylglycerol
DGDG: Digalactosyldiacylglycerol
EPA: Eicosapentaenoic acid
ELOVL4: Elongation of very long chain fatty acids protein 4
ELOVL6: Elongation of very long chain fatty acids protein 6
ETNPPL: Ethanolamine-phosphate phospho-lyase
FA: Fatty acyl class
FAMEs: Fatty acid methyl esters
FLNC: Filamin C
FPKM: Fragments per kilobase of transcript per million mapped reads
GAPDH: Glyceraldehyde-3-phosphate dehydrogenase
GL: Glycerolipids
GP: Glycerophospholipids
GPAT3: Glycerol-3-phosphate acyltransferase 3
GSH-Px: Glutathione peroxidase
IMF: Intramuscular fat
KEGG: Kyoto encyclopedia of genes and genomes
L*: Lightness value
LD: Longissimus dorsi
MAPK: Mitogen-activated protein kinase
MDA: Malondialdehyde
MOGAT2: Monoacylglycerol O-acyltransferase 2
MUFA: Monounsaturated fatty acid
OPLS-DA: Orthogonal partial least squares discriminant analysis
PC: Phosphatidylcholine
PCA: Principal component analysis
PE: Phosphatidylethanolamine
PISD: Phosphatidylserine decarboxylase
PLD4: Phospholipase D family member 4
PR: Prenol lipids
PUFA: Polyunsaturated fatty acid
qRT-PCR: Quantitative real-time reverse transcription PCR
RLBP1: Retinaldehyde binding protein 1
SCD: Stearoyl-CoA desaturase
Se: Selenium
SEM: Standard error of the mean
SFAs: Saturated fatty acids
SGMS2: Sphingomyelin synthase 2
GlcSph: Glucosylsphingosine
SP: Sphingolipids
SQMG: Sulfoquinovosylmonoacylglycerol
ST: Sterol lipids
T-AOC: Total antioxidant capacity
TG: Triglyceride
T-SOD: Total superoxide dismutase
