# Supplementary material for: Se-enriched yeast improves meat quality through glycerophospholipid metabolism in finishing pigs: insights from a multi-omics analysis
Source: J Anim Sci Biotechnol. 2026 Jul 5;17:139. doi: 10.1186/s40104-026-01446-3 (PMC13333091; doi:10.1186/s40104-026-01446-3)
Supplement: Supplementary file 2 — Additional file 2: Fig. S1. Lipidomic analysis of longissimus dorsi muscle in CT, SeD, and SY3 groups. Fig. S2. Transcriptomic analysis of longissimus dorsi in SeD, CT, and SY3 groups. [file 40104_2026_1446_MOESM2_ESM.docx]

**
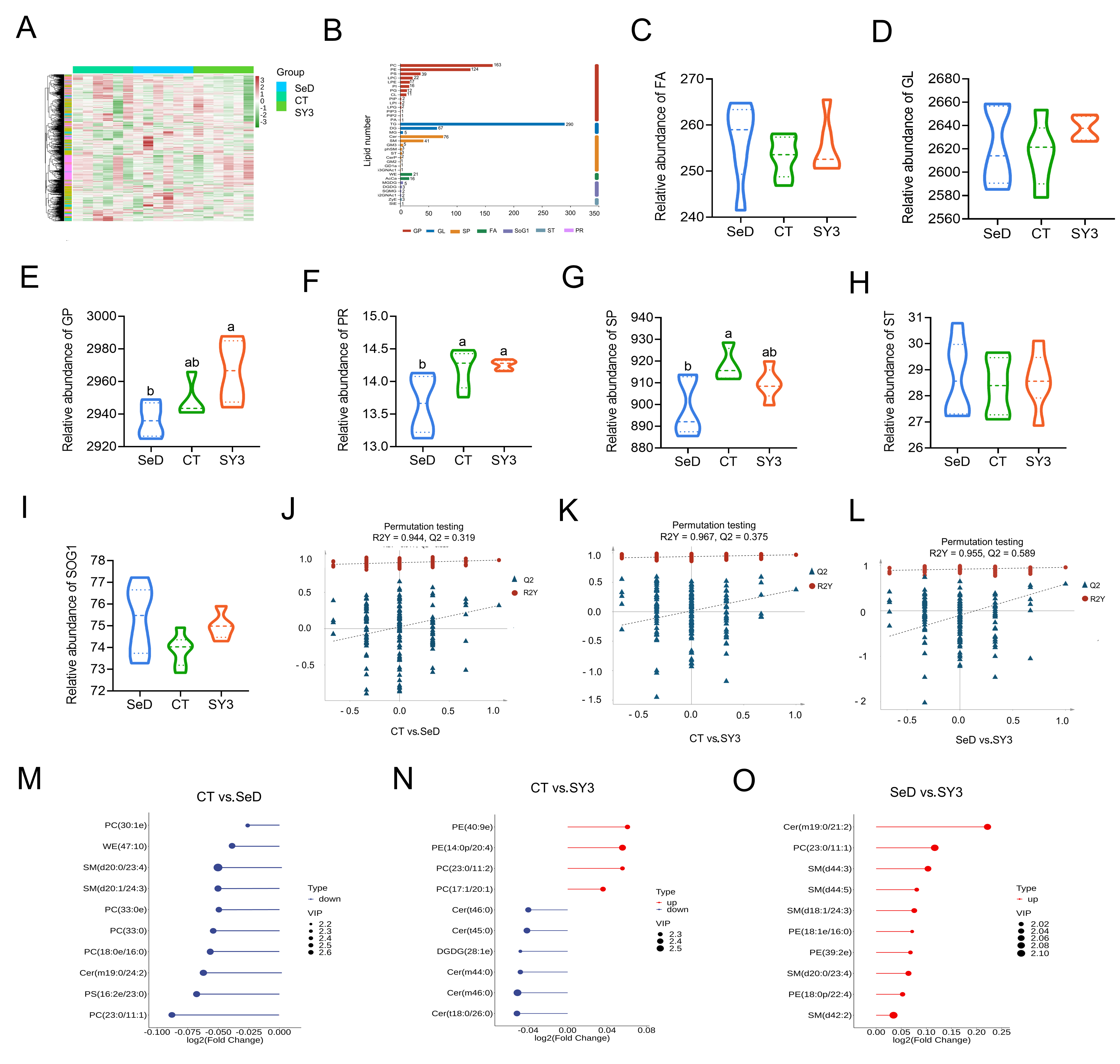
Fig. S1** Lipidomic analysis of longissimus dorsi (LD) muscle in CT, SeD, and SY3 groups. **A** Heatmap of lipidomic profiles. **B** Distribution of lipid species in 34 lipid subclasses. **C–I** Relative abundance of seven classes of lipids. **J–L** OPLS-DA models validated by permutation tests in three pairwise comparisons; **M–O** Representative DELs of LD muscle in three pairwise comparisons. SeD, Se-deficient group; CT, control group (basal diet); SY3, Se-enriched yeast group (basal diet + 3 mg Se/kg). Data were presented by mean ± SEM, *n* = 6. Different letters indicate significant difference (*P* < 0.05)


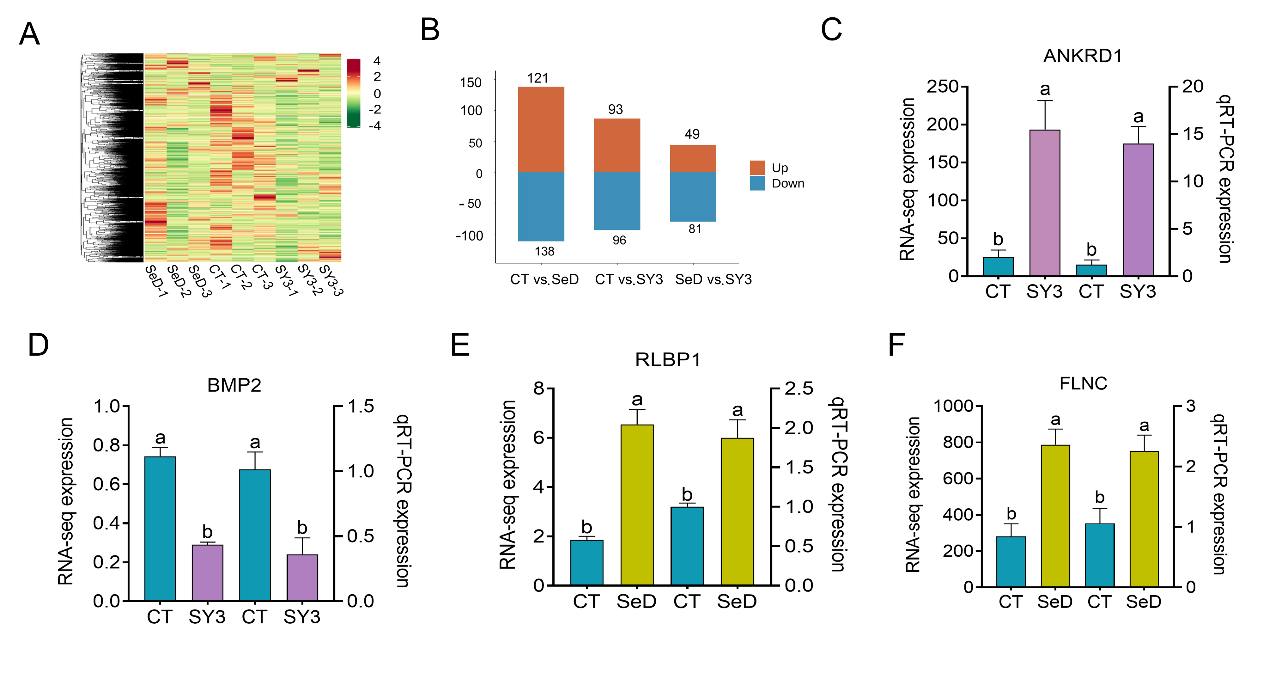


**Fig. S2** Transcriptomic analysis of longissimus dorsi (LD) in SeD, CT, and SY3 groups.

**A** Heatmap of global gene expression profiles. **B** Number of differentially expressed genes (DEGs) of LD in three pairwise comparisons. Orange bars indicate upregulated genes; blue bars indicate downregulated genes. **C–F** Verification of DEGs by RT-qPCR. SeD, Se-deficient group; CT, control group (basal diet); SY3, Se-enriched yeast group (basal diet + 3 mg Se/kg). Data were presented by mean + SEM, *n* = 6. Different letters indicate significant difference (*P* < 0.05)
