# Supplementary material for: Se-enriched yeast improves meat quality through glycerophospholipid metabolism in finishing pigs: insights from a multi-omics analysis
Source: J Anim Sci Biotechnol. 2026 Jul 5;17:139. doi: 10.1186/s40104-026-01446-3 (PMC13333091; doi:10.1186/s40104-026-01446-3)
Supplement: Supplementary file 3 — Additional file 3. The uncropped Western blot images. [file 40104_2026_1446_MOESM3_ESM.docx]

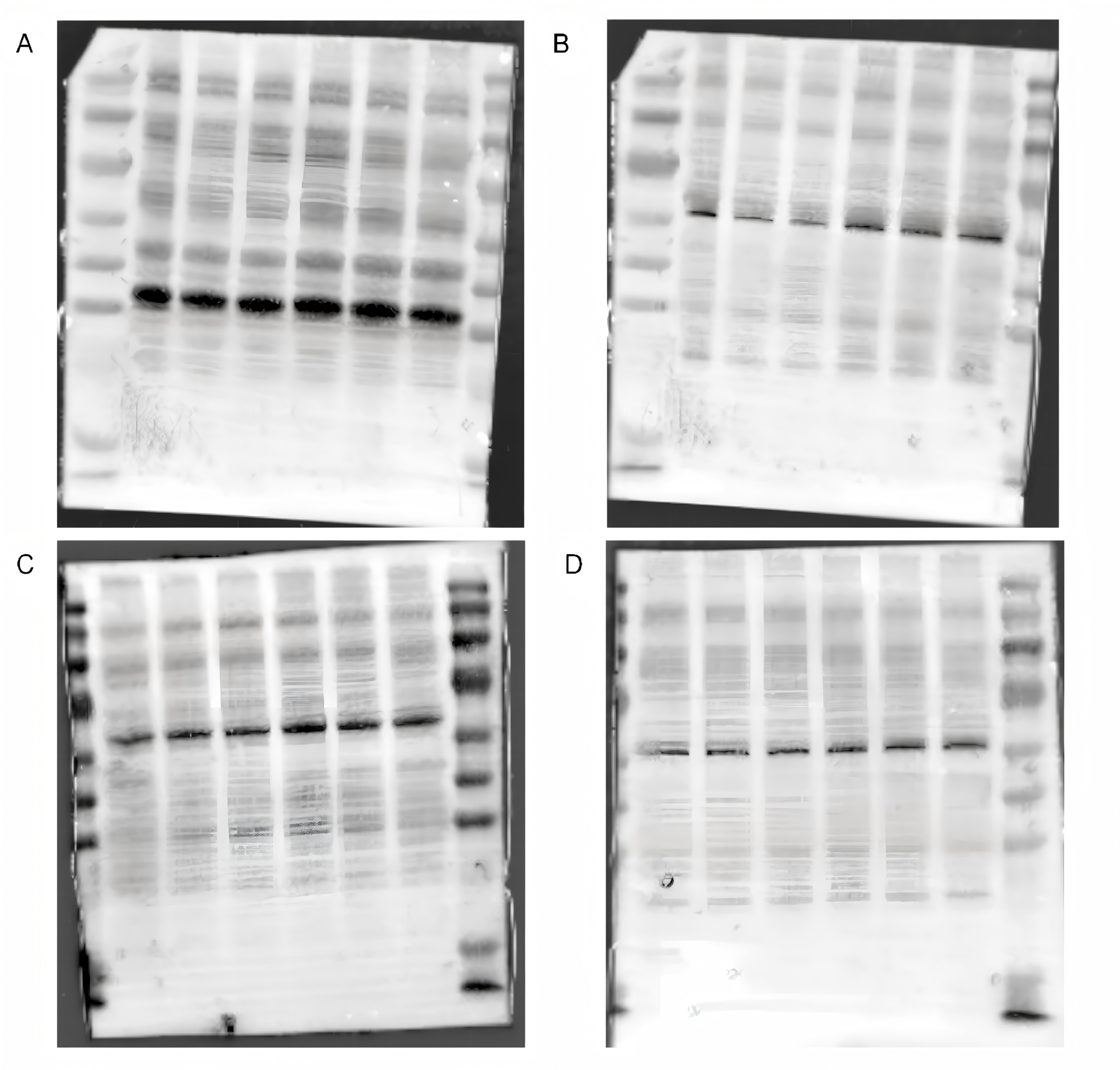


**Fig. 1.** The uncropped Western blot images.

**A**: Western blot analysis of GAPDH; **B**: Western blot analysis of GPAT3 in the CT and SY3 groups; **C**: Western blot analysis of PISD in the SeD and CT groups; **D**: Western blot analysis of GPAT3 in the SeD and CT groups
